# Successful Treatment of Localized Cutaneous Melioidosis with Oral Antibiotic Therapy Alone

**DOI:** 10.4269/ajtmh.18-0940

**Published:** 2019-05

**Authors:** Simon Smith, Josh Hanson

**Affiliations:** 1 Department of Medicine, Cairns Hospital, Cairns, Australia;; 2 James Cook University, Cairns, Australia;; 3 The Kirby Institute, New South Wales, Australia

**Figure 1.**
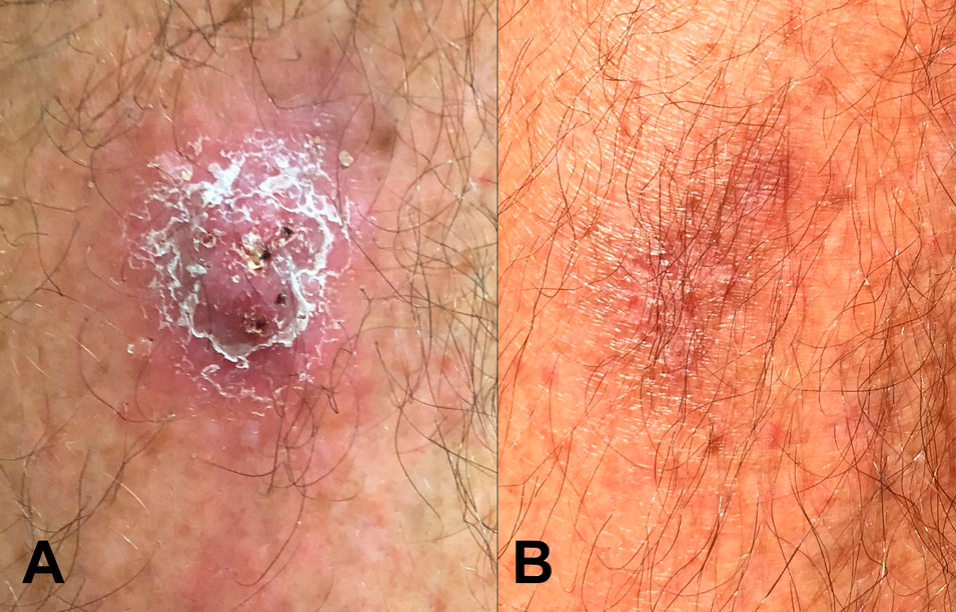
(**A**) Localized skin lesion caused by melioidosis. (**B**) Complete resolution of the lesion after 3 months of oral antibiotic therapy alone. This figure appears in color at www.ajtmh.org.

A 65-year-old man, who was usually well, presented to the outpatients’ department of Cairns Hospital, in tropical Australia with a 3-week history of an evolving, but non-painful, purulent lesion on his right shin. He was afebrile and had no other symptoms. He enjoyed gardening and recalled sustaining multiple superficial skin lacerations and being exposed to flood water approximately 8 weeks previously. His physical examination was completely unremarkable, apart from a single raised, erythematous, indurated, crusted, 2 × 2-cm lesion on his right shin (Figure 1A). A superficial swab of the lesion isolated *Burkholderia pseudomallei*, establishing the diagnosis of melioidosis. Two sets of blood cultures were negative, and a chest X-ray and computerized tomography scan of the abdomen and pelvis were normal. It was recommended that the patient receive outpatient intravenous ceftazidime through a peripherally inserted central venous catheter; however, he was concerned about the potential for catheter-associated complications, and elected to receive only oral therapy. He was therefore treated with a 3-month course of trimethoprim/sulfamethoxazole (TMP/SMX), 320/1,600 mg twice daily, with daily folic acid and close outpatient monitoring. He noticed significant improvement after only 2 days of therapy, and the lesion had resolved completely by the end of treatment (Figure 1B). Two months later, there has been no evidence of recurrence.

Melioidosis is a disease of public health importance in endemic areas, particularly Southeast Asia and northern Australia. Current Darwin guidelines recommend that localized, cutaneous melioidosis should be treated with a minimum 2-week intensive phase of intravenous meropenem or ceftazidime plus concurrent oral TMP/SMX, followed by a 3-month oral eradication phase with oral TMP/SMX.^1^ There have been no clinical trials evaluating the efficacy and safety of oral therapy alone for the treatment of localized, cutaneous melioidosis; however, cure has been documented with oral therapy alone.^2,3^ Approximately 13–24% of the estimated 165,000 global cases of melioidosis each year will affect only skin and soft tissue.^4^ Entirely oral treatment could be considered in individual cases of localized, cutaneous disease, particularly in immunocompetent patients. However, appropriate cultures and imaging should be performed on all patients to exclude disseminated infection, which can be fatal in the absence of appropriate therapy.^5^
